# The Role of Gastrointestinal Dysbiosis and Fecal Transplantation in Various Neurocognitive Disorders

**DOI:** 10.7759/cureus.72451

**Published:** 2024-10-26

**Authors:** Zainab A Castro-Vidal, Felwin Mathew, Alia A Ibrahim, FNU Shubhangi, Robin R Cherian, Hoi Kei Choi, Afreen Begum, Hari Krishna Ravula, Harshvardhan Giri

**Affiliations:** 1 Surgery, Saba University School of Medicine, The Bottom, BES; 2 Neurology, PK Das Institute of Medical Science, Ottapalam, IND; 3 Internal Medicine, Dr. Sulaiman Al-Habib Hospital - Al Sweidi Branch, Riyadh, SAU; 4 Internal Medicine, Nalanda Medical College and Hospital, Patna, IND; 5 Medicine, Kasturba Medical College, Manipal, Manipal, IND; 6 Psychology/Neuroscience, University of Michigan, Ann Arbor, USA; 7 Medicine, Employee State Insurance Corporation (ESIC) Medical College and Hospital, Hyderabad, IND; 8 Medical Education, Dhaka Medical College and Hospital, Dhaka, BGD; 9 Bioengineering, University of California, Los Angeles, USA

**Keywords:** alzheimer's disease, dysbiosis, fecal microbiota transplantation, gut-brain axis, microbiome, multiple sclerosis, neurological disorders, parkinson's disease

## Abstract

This review explores the critical role of the human microbiome in neurological and neurodegenerative disorders, focusing on gut-brain axis dysfunction caused by dysbiosis, an imbalance in gut bacteria. Dysbiosis has been linked to diseases such as Alzheimer's disease, Parkinson's disease (PD), multiple sclerosis (MS), and stroke. The gut microbiome influences the central nervous system (CNS) through signaling molecules, including short-chain fatty acids, neurotransmitters, and metabolites, impacting brain health and disease progression. Emerging therapies, such as fecal microbiota transplantation (FMT), have shown promise in restoring microbial balance and alleviating neurological symptoms, especially in Alzheimer's and PD. Additionally, nutritional interventions such as probiotics, prebiotics, and specialized diets are being investigated for their ability to modify gut microbiota and improve patient outcomes. This review highlights the therapeutic potential of gut microbiota modulation but emphasizes the need for further clinical trials to establish the safety and efficacy of these interventions in neurological and mental health disorders.

## Introduction and background

The overwhelming majority of the one million to one trillion microorganisms that comprise the human ecosystem live in the human digestive tract. When discussing human digestive systems, the phrase "gastrointestinal tract microbiota" is used to describe the whole bacterial genome [1]. The gastrointestinal tract and the brain axis engage in mutually beneficial interactions. A disturbance in the microbiome's delicate equilibrium, known as dysbiosis, may contribute to the onset of neuropsychiatric diseases such as multiple sclerosis (MS), Alzheimer's disease (AD), depression, anxiety, and Parkinson's disease (PD) [2].

Dysbiosis is defined as an unbalanced microbiome that lacks certain good and bad bacteria, as well as the metabolites and antigens that these bacteria produce [3]. Gut bacteria may interact with the central nervous system (CNS) in several ways despite the fact that the brain and the stomach are biologically distinct. Hormones, metabolites, and neuroactive chemicals are all byproducts of these functions, and they influence many bodily systems, including the parasympathetic vagus nerve, the immune system, the endocrine system, the gastrointestinal system, and the circulatory system [4]. Potential effects on brain function may result from bacterial waste products in the digestive tract, which include lactate, bile acids, and neurotransmitters such as acetylcholine. Disorders of the nervous system may develop when there is discordance between these systems [5].

Innovative treatment modalities that target the gut microbiota to alleviate neurological disease symptoms have emerged due to our growing understanding of the gut-brain axis (GBA) [6]. The innovative fecal microbiota transplantation (FMT) procedure helps patients with dysbiosis. This method aims to restore or maintain a stable environment for the host and its internal bacteria by transferring healthy microbiota from a donor to a recipient. Crucially, the outcome seems unaffected by the nature of the relationship between the patient and the donor [7]. The gut microbiota provides fascinating and undervalued insights into the development of several diseases. The underlying idea is that the microbiota in our gut and the chemicals it generates play a key role in keeping our gut in a state of homeostasis. These compounds have complex pathophysiological pathways that provide the groundwork for creating therapeutic methods such as FMT [8]. The relationship between the gut flora, the CNS, and modern medicine has received a lot of attention lately. The increasing amount of evidence pointing to this link implies that it will emerge as a significant field of study in the realm of healthcare and nutritional practices in the future [9]. The issue has garnered attention due to the mounting evidence of FMT's therapeutic importance. The importance of FMT as a therapy for neurocognitive disorders will be discussed after an evaluation of the function of gut dysbiosis in the development of these illnesses.

## Review

Neurodegenerative diseases

Alzheimer’s Disease

Alois Alzheimer, a German psychiatrist, was the inspiration for AD, the most common kind of dementia. Neurofibrillary tangles and neural plaques are the defining characteristics of this neurodegenerative condition, which is often characterized as a slowly progressing disease. The outer layer of the neocortex and the middle section of the temporal lobe are the areas most affected. Both the cholinergic and the amyloid hypotheses provide compelling explanations for how AD progresses. Some of the genetic anomalies that increase the likelihood of this disorder include mutations in the amyloid precursor protein (APP) and presenilin-1 (PSEN-1)/presenilin-2 (PSEN-2), as well as age and several environmental factors and microlevel deficiencies [10].

Gut Microbiota-Brain Axis (GMBA) and AD

The GMBA has recently attracted a lot of interest and scrutiny from the scientific community. Because of this, it is now considered a viable therapeutic target for several diseases affecting the CNS, such as AD. Metabolic, immunological, and neuroendocrine pathways are just a few of the numerous ways that research shows that gut microbiota affects CNS function [11]. The microbiota produces signaling molecules such as tryptophan, lipopolysaccharides (LPS), leptin/ghrelin hormones, and short-chain fatty acids in the intestines. These substances influence the CNS [12]. The anti-AD effects of short-chain fatty acids (SCFAs) are largely due to their ability to activate G-protein-coupled receptors, influence cell proliferation, and change histone acetylation. Furthermore, it has been shown that persons with AD do not contain enough SCFAs [13].

LPS have a dual purpose in the progression of AD. Research using animal injections has shown that they impair cognition by stimulating the production of amyloid-β proteins in the hippocampal area, thereby adding to the amyloid hypothesis. The production of pro-inflammatory cytokines and immunoglobulins (M and A) results from an immunological pathway that LPS activate; however, this pathway causes systemic inflammation. As previously mentioned, this verifies the immunological route [14].

Evidence suggests that AD patients experience a shift in gut flora toward more inflammatory forms of bacteria rather than anti-inflammatory ones. For instance, as we get older, which is known to increase the likelihood of AD, certain bacteria such as *Bacillus fragilis*, *Bacteroides fragilis*, *Faecalibacterium prausnitzii*, *Eubacterium hallii*, and *Eubacterium rectale *proliferate, and other bacteria with anti-inflammatory characteristics become less common. According to research, certain amyloid-producing bacteria directly generated the production of amyloid-β oligomers (AβO)/fibrils [14] in the CNS of mouse models. These bacteria include *Escherichia coli*, *Salmonella enterica *serovar Typhimurium, *Bacillus subtilis*, *Pseudomonas fluorescens*, and *Staphylococcus aureus*. In light of this, the amyloid hypothesis proposes that gut microbiota may have a role in AD development. In contrast to healthy persons, Cattaneo et al. discovered pro-inflammatory microbes in amyloidosis patients [13].

Because of their diversity in gasotransmitters produced by the brain, the gut microbiota plays an additional role in AD development. Nitrous oxide, carbon monoxide, ammonia, hydrogen sulfide, and methane are common chemicals that fall within this category [15]. The majority of ammonia is generated in the gastrointestinal tract by microbes via the amino acid deamination process and the breakdown of urea. According to Cooper and Plum, the neurotoxic effects of elevated ammonia concentrations have been associated with Alzheimer's dementia. According to Adlimoghaddam et al., the blood-brain barrier is changed by ammonia, and astrocyte/neuronal structures are altered [16]. Ammonia also causes depolarization of neuronal action potentials in the hippocampus. Along with nitric oxide and carbon monoxide, hydrogen sulfide has recently been acknowledged as the third major gasotransmitter. The onset of AD has also been linked to it [17].

**Figure 1:**
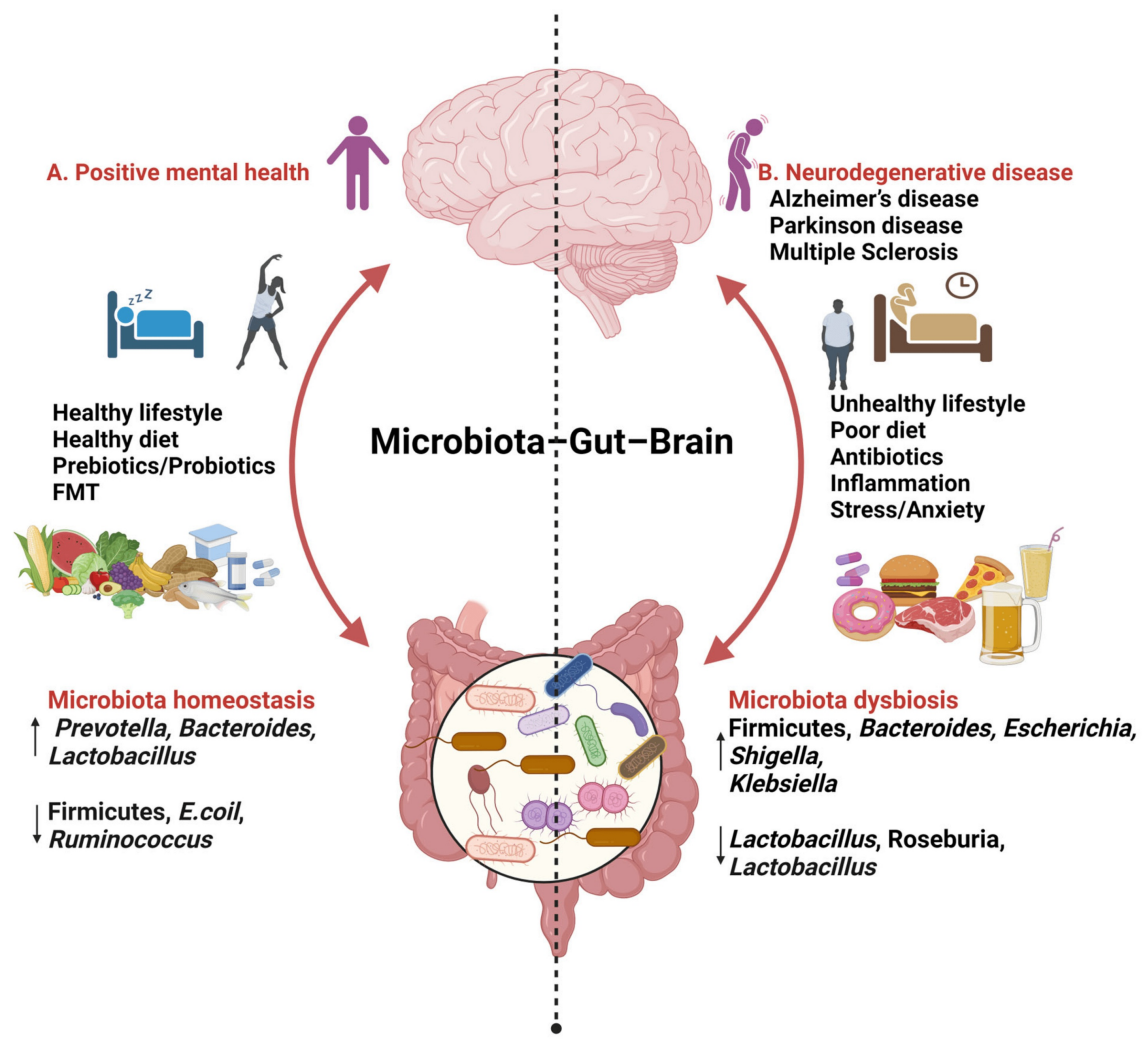
Gut–brain–microbiota interactions Reproduced under the terms and conditions of the Creative Commons Attribution (CC BY) license (https://creativecommons.org/licenses/by/4.0/) from reference [22]. Copyright © 2024 by the authors. Licensee MDPI, Basel, Switzerland.

The gut microbiota has been linked to AD via its effects on tryptophan, a signaling molecule in the GBA and an essential building block for several neurotransmitters in the brain. The first is to consume tryptophan orally, which may limit the host's access to it. More than that, the tryptophanase enzyme allows certain bacterial strains to convert tryptophan into indoles [18]. The first is that these indoles boost the growth of the gut microbiota in many ways, including drug resistance, spore formation, virulence, biofilm structure, and plasmid stability [19]. The second is that they directly affect the neurotransmitters acetylcholine and serotonin, as well as other tryptophan metabolites produced by bacteria [20]. The gut microbiota indirectly impacts neurotransmitters via the formation of AβO/fibrils in the brain. Cholinergic, serotonergic, and GABAergic brain cells may be poisoned by these oligomers, causing them to stop producing crucial brain chemicals [21]. This alteration, in turn, affects the GBA, potentially leading to the alleviation of AD-related pathology (Figure 1) [22].

FMT and AD

There is recognition of the use of FMT in mouse model research to examine the possibility that germ-free mice may develop CNS problems after ingesting fecal material from AD animals. Research using the transplantation of fecal contents from AD-expressing animals onto healthy mice serves as an example. Deposition of brain plaques, poor neurogenesis, elevated levels of pro-inflammatory cytokines, decreased BDNF levels, and worsened memory impairment were all outcomes of this transplantation [14]. Moreover, germ-free mice were administered FMT from senescence-accelerated mice (SAM) and senescence-accelerated mice resistant (SAMR). These mice already exhibited significant cognitive impairment prior to the experiment. Researchers employ two mouse strains as models: SAM, which shows signs of accelerated aging, and SAMR, which shows signs of normal aging. Brain tests performed far better in germ-free mice with FMT from SAMR mice than in the SAM animals. The results show that older SAM have cognitive loss due to certain microorganisms [11]. Furthermore, an increased buildup of plaques was observed when APPPS1 transgenic mice, which carry human transgenes for both the *APP* gene (which causes familial AD) and the *PSEN1* gene (which causes early-onset AD), were transferred to GF mice through FMT [23]. Fascinatingly, FMT in germ-free mice caused an increase in cognitive decline and a decrease in microbiota-produced metabolites that are important for CNS function [24]. AD patients' feces were used in this study.

FMT has shown promise as a therapy for AD in several trials. For example, one study employed AD mouse models to transplant healthy fecal microbiota from inbred wild-type mice. This led to a clinical improvement in cognitive impairment, a decrease in pro-inflammatory markers, and a decrease in amyloid buildup. Improvements in cognitive performance and reductions in amyloid accumulation were also verified in another study. There was also an improvement in synaptic plasticity, which means that connections may become stronger or weaker in response to variations in activity. In addition to an increase in the proliferation of gut microbiota that generate SCFAs, the research also discovered a decrease in tau protein expression [25]. However, regarding people, there have only been two verified cases of successful outcomes. In the first study, an 82-year-old guy had FMT from a female donor who was 85 years old. Getting FMT (from a 27-year-old boy) to treat *Clostridioides difficile* infection was reported in the second case report as an experience of a 90-year-old female with a known history of AD for five years [26,27].

Although FMT has shown promise in mice, more research in people is needed to confirm its efficacy as a treatment for AD. Additionally, for optimal results, it is important to define administration protocols, including methods, therapeutic duration, risk stratification, and inclusion/exclusion criteria [28]. Despite the lack of strong and clear conclusions, recent studies examine the impact of prebiotics, probiotics, and dietary approaches such as the Mediterranean, Dietary Approaches to Stop Hypertension, and ketogenic diets for preventing and treating AD. These trials provide quite encouraging outcomes [14].

Dementia with Lewy bodies (DLB)

The aberrant buildup of α-synuclein fibrils, mostly in the limbic regions and sometimes in the neocortex, is the hallmark of the well-documented neurodegenerative disease DLB. Visual hallucinations, parkinsonism (movement irregularities comparable to PD), autonomic dysfunctions, sleep difficulties, cognitive impairment (ranging in severity), and dementia are the hallmarks of this syndrome [29]. There are similarities between PD and degenerative limb wasting, two neurodegenerative diseases defined by the buildup of Lewy bodies. On the other hand, parkinsonism symptoms often appear later in DLB compared to PD, whereas dementia tends to emerge sooner [30]. When there are no other differentiating features of DLB, the parallels between DLB and AD become even more apparent. In addition to the buildup of Lewy bodies, additional pathological changes such as those of AD are also present in DLB. Diagnosing them could be difficult due to the similarity in their underlying disorders and clinical symptoms [31].

GMBA and DLB

In DLB, the bacteria responsible for producing SCFAs decrease in number, significantly impacting these acids' neuroprotective qualities. New evidence suggests that people with DLB have significantly reduced populations of seven different types of gut bacteria. It was discovered that six of these bacteria could create SCFAs [32]. In addition, in relation to AD, all cases of Lewy body dementias (LBDs) were associated with an increase in LPS, a substance known to have neurotoxic effects. The pathogenic consequences of AD and DLB are quite similar. Some fibrils clump together, e.g., Aβ-protein fibrils in AD and α-synuclein fibrils in dementias lingering after brain damage. Systemic inflammation is another symptom shared by the two disorders [33].

According to the aforementioned study, the same people were also found to have high levels of two particular bacteria, *Collinsella* and *Ruminococcus torques*. The capacity to halt the death of dopamine-producing cells in the substantia nigra induced by neurotoxicity was shown to be possessed by these organisms due to their excessive production of ursodeoxycholic acid (UDCA). Suppressing certain inflammatory cytokines accomplishes this. The delayed start of parkinsonism movements in DLB, a distinguishing clinical feature between DLB and PD [32,34], may be explicable by the lack of these two microorganisms in the PD subjects of the same study. Consequently, it is also thought that countries where the *Collinsella* bacteria are abundant have lower COVID-19 mortality rates. The anti-inflammatory effects of UDCA have already been mentioned, which might explain this. Ryman et al. proposed two theories that built on Braak et al.'s idea that a vagus nerve-borne gut infection might be the root cause of PD. The first theory proposes that α-synuclein initially aggregates in the intestines before reaching the CNS. The second theory states that Lewy body disorders arise due to inflammatory processes that start in the gastrointestinal system. The gut microbiota largely impact both ideas [35].

FMT and DLB

Research in that particular field is currently lacking. Further investigation into the potential use of FMT for other neurodegenerative illnesses, such as DLB and other LBDs, is encouraged by the current research on FMT in relation to AD, MS, and mental problems.

Parkinson's disease

Tremors, bradykinesia, and postural instability are motor symptoms of PD, a complex neurodegenerative disorder. The role of the gut microbiota in PD onset and progression is becoming increasingly apparent in research. There is a strong correlation between the GBA, a two-way communication connection between the gastrointestinal system and the CNS, and the impact of gut microbes on neurological health. People suffering from PD often exhibit gut microbiome changes, marked by an overabundance of inflammatory bacteria and a deficiency of beneficial microbes. Modifications to the gut microbiome's microbial makeup in PD patients point to a major role in the disease's onset. Since changes in the gut microbiota manifest early in the course of PD, this may have some bearing on the beginning of neurological impairment. Scientists are, therefore, compelled to probe the connection between these two occurrences. New evidence has linked the gut microbiome to PD progression and development [36]. Researchers found that the gut microbiome of people with PD is out of whack, with an overabundance of bacteria associated with inflammation and disease-causing microbe. Researchers also found that the gut microbiome of people with PD exhibited an overabundance of certain genes and metabolic pathways. These results indicate a major role for the gut flora in PD development [36].

In the setting of PD and its symptoms, recent research has focused on the complex relationship between the gut flora and the brain. Through these studies, we want to learn more about how PD symptoms are related to the brain's and the gut's interplay. A possible correlation between the onset of neurological symptoms and dysbiosis in the gut has been suggested by studies showing alterations in the gut microbiome in pre-disease phases of PD and REM (rapid eye movement) sleep behavior disorder (RBD) [37,38]. Reducing butyrate-producing bacteria and increasing pro-inflammatory *Collinsella* in RBD patients is comparable to the pre-clinical symptoms of PD, which include an imbalance in gut microbial composition. In dysbiosis, the gut's balance of good and bad bacteria is upset. Increased intestinal permeability, immune system activation, and the development of α-synuclein in the enteric nervous system are some of the detrimental repercussions that this imbalance produces [39,40]. This condition highlights the crucial role of the GBA in developing PD since the abnormal accumulation of α-synuclein in the enteric nervous system usually happens before any central neurodegeneration and subsequently moves to the CNS in PD [39,41].

According to research [42], there is a link between gut dysfunction and the early phases of PD since patients often have gastrointestinal symptoms prior to any motor impairment. Extensive studies on the gut microbiome have shown that it plays a significant role in regulating and influencing neurodegenerative illnesses such as PD. In particular, they have demonstrated that PD patients' gut microbiome abnormalities might lead to systemic inflammation, which in turn can exacerbate gastrointestinal symptoms. Moreover, alterations in the gut microbiota are associated with PD symptoms and PD etiology. This further proves that PD treatments aimed at the gut microbiota might be effective [39,42]. Additional information on the GBA's role in PD inflammation and α-synuclein buildup may be gleaned from animal research. This deepens our understanding of PD and opens new therapeutic possibilities [42].

A key component in the development of PD is the reciprocal relationship between the CNS and gut microbiota, which is called the brain-gut-microbiome-immune axis. The complex relationship between gut microbiota, immunological responses, and neurodegenerative changes in PD is suggested by the brain-gut-microbiome-immune axis [42]. Several studies from various countries have shown comparable patterns of alterations in the gut microbiota in people with PD. Similar findings in other inflammatory gastrointestinal diseases and neurological illnesses support the identification of changes, such as changes in microbiota composition. These shifting patterns further support the role of gut microbiota in PD development [40]. These results show that the gut microbiota may be a target for therapies aimed at reversing the neurodegenerative effects of PD, which is a complicated disorder.

Medicinal modification of the gut microbiota is undoubtedly an exciting and cutting-edge area of research into PD treatment. For the time being, most studies investigating potential treatments for PD symptoms have focused on altering the composition of the gut flora. A key regulator of PD progression and development is the GBA, which permits two-way communication between the gastrointestinal and central neurological systems. Intestinal permeability, neuroinflammation, and neurotransmitter production are three ways in which the GBA affects disease progression [39]. Therefore, in order to improve diagnostic tools and therapeutic options, such as the use of probiotics, prebiotics, or targeted dietary alterations, it is essential to understand how changes in the gut microbiota affect PD symptoms [42].

**Figure 2:**
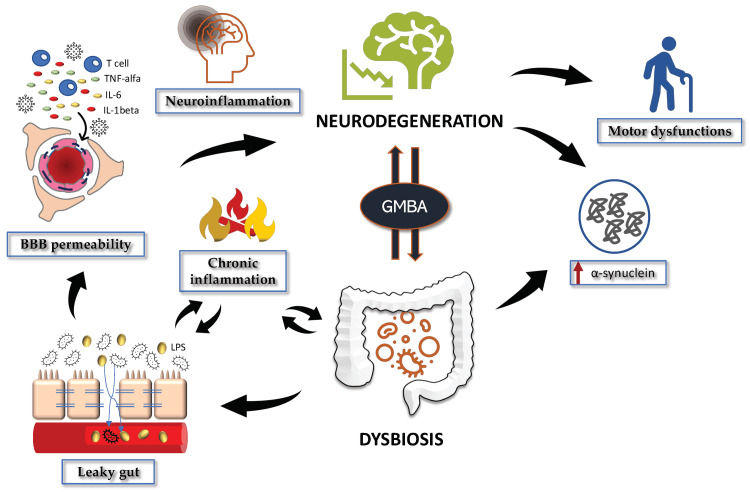
The gut–microbiota–brain axis (GMBA) in Parkinson's disease (PD) BBB: blood-brain barrier; TNF-alpha: tumor necrosis factor-alpha; IL-6: interleukin-6; IL-1beta: interleukin-1 beta; GMBA: gut–microbiota–brain axis. Reproduced under the terms and conditions of the Creative Commons Attribution (CC BY) license (https://creativecommons.org/licenses/by/4.0/) from reference [43]. Copyright © 2022 by the authors. Licensee MDPI, Basel, Switzerland.

There is a complex relationship between gut health and PD symptoms since the composition of the gut microbiota is affected by personal characteristics, including age, gender, and drug history. Ultimately, these factors highlight the GBA's major role in the development and course of PD, opening the door to new avenues of investigation and potential treatments. In relation to PD, dysbiosis and the accompanying gastrointestinal symptoms and gut discomfort occur well before the appearance of motor dysfunctions. This is associated with neuroinflammation and changes in the metabolism of dopamine, serotonin, and kynurenine via the GBA (Figure 2) [43].

Multiple sclerosis and the GBA

The autoimmune disease MS causes demyelination and neuroinflammation, which in turn cause a variety of neurological symptoms. According to the research, there is strong evidence linking the gut microbiome to MS onset. Neuronal connections, general neuroendocrine and humoral pathways, and the immune system are the three main avenues by which the gut microbiota may impact the brain. The vagus nerve and the enteric nervous system are the principal neural routes to the gastrointestinal tract. Metabolites and the microbiota in MS patients' guts differ from those of healthy people. According to research, MS is believed to be significantly aided by chemicals derived from the gut microbiota[44]. Several metabolites, including bile acid receptors, medium-chain fatty acids, very long-chain fatty acids, and SCFAs, have been shown to significantly impact neuroinflammation and immune system activation. T helper cells, regulatory T cells, interleukin, cerebrospinal fluid, experimental autoimmune encephalomyelitis, and the blood-brain barrier are all involved in these processes [45].

There are several paths that serotonin, acetylcholine, serotonin derivatives, and precursors take from the stomach to the brain. These include the vagus nerve, circulation, the HPA (hypothalamic-pituitary-adrenal) axis, and induction. The CNS is affected by these metabolites by many mechanisms, including changes in intestinal permeability, induction of proinflammatory cytokines, disruption of the blood-brain barrier, and anti-inflammatory effects [46]. This is crucial because the development and treatment of MS will be drastically changed if we can provide metabolites to make more beneficial metabolites in MS patients. Compared to healthy donors, the microbiota of MS patients shows substantial variance. MS patients were shown to have a decreased prevalence of *Bacteroides*, *Parabacteroides*, *Prevotella*, *Sutterella*, and *Lactobacillus* but a higher prevalence of *Akkermansia*, *Ruminococcus*, *Blautia*, and *Bifidobacterium* [45]. *Ruminococcus torques*, *Ruminococcus obeum*, *Lactospiraces*, *Escherichia coli*, and *Oscillibacter *were found to be more prevalent in a separate case-control study. In contrast, *Bacteroides fragilis*, *Roseburia*, *Haemophilus parainfluenzae*, and *Sutterella wadsworthensis* were shown to be less prevalent [47].

New treatment modalities have been developed as a consequence of our growing knowledge of the gut microbiota difference between healthy persons and those with MS. Dietary changes, probiotic treatment, frequent exercise, fecal transplants from donors with a healthy microbiota, and similar approaches are used to alter the gut microbiota's makeup. There is evidence that people with MS may benefit from a combination of *Lactobacilli*, *Limosilactobacillus reuteri*, and bifidobacteria [48]. The capacity of vancomycin to promote the growth of microbes that activate T regulatory cells is probably responsible for its efficacy when given intravenously [49].

Research into the effects of gut microbiota on MS is an exciting new area with great therapeutic potential. With further knowledge of how the microbiome and metabolites affect immunological and neuroinflammatory disorders, new methods for treating and preventing MS could be possible.

Stroke

According to the World Health Organization [50], stroke is a major public health issue on a worldwide scale since it is the leading cause of death and disability in the globe. Every year, almost 15 million people have a stroke. Out of them, five million will lose their lives, and another five million will be permanently disabled. This study used essential fatty acids to identify 15 metabolite variables associated with certain metabolic pathways. Both the fully adjusted and base models showed that factor 3, out of the 15 metabolite variables studied, was associated with an increased risk of ischemic stroke. Digestive microbiota-related chemicals improved this variable [51]. The severity of strokes [52], increased risk of cardiovascular disease [53], and atherosclerosis have all been linked to trimethylamine N-oxide (TMAO) in previous research. Precursors that include trimethylamine, such as phosphatidylcholine, and choline, which are often consumed in Western diets, are broken down by microbes. TMAO is produced and circulated due to this metabolism [53]. A Western diet, especially heavy in red meat, dramatically raises TMAO levels [54,55]. Stroke causes a metabolic shift towards catabolism, which in turn causes a considerable loss of muscle mass and body weight, according to studies done on animal models and in human trials. According to studies, these variations in body weight may be linked to gut flora variations [56].

Possible neuropsychiatric disorders treatable with microbes

Twenty years after gut microbiota and microbial-gut-brain (MGB) investigations on cognitive function showed hopeful findings, alternative strategies concentrating on gut dysbiosis in neurodevelopmental disorders are gaining popularity. Extensive evidence suggests that gut dysbiosis significantly affects anxiety and behaviors associated with mood [57,58]. New research has proven beyond a reasonable doubt that changes in the diversity, stability, composition, and maintenance of gut microbes negatively impact host health and make them more susceptible to a wide range of non-communicable diseases, some of which can be quite severe [59,60]. Numerous synthetic pharmacological substances are used to address various NPDs in human patients; nevertheless, their effectiveness differs when tested in clinical environments, and the adverse effects usually outweigh the benefits. More and more investigations are corroborating the results of preclinical trials. According to this study, eubiosis, or the promotion of a balanced microbial composition and variety, seems to be the key to reestablishing a healthy gut. Incorporating dietary adjustments, fasting, calorie restriction, and supplementation with probiotics, prebiotics, synbiotics, or FMT are some of the ways to achieve this goal [61,62]. Prebiotics, probiotics, and synbiotics are examples of novel medicines that target triggers, and research on these products has shown therapeutic advantages in patients with neurological illnesses [63]. Thus, our findings suggest that improving gastrointestinal and psychiatric symptoms in NPD patients may be possible by addressing MGB axis signaling.

Probiotics

Consumption of live microbes that encourage the growth of beneficial bacteria species is known as a probiotic or probiotic combination [64]. Probiotics with improved neuroprotective properties are known as psychobiotics, and they find widespread use in the treatment of neurodevelopmental disorders [65]. Patients with MS, cognitive impairments, and stress-related disorders have shown beneficial results after receiving lactic acid bacteria and bifidobacteria as a treatment [66,67]. It is widely acknowledged that some bacterial strains, such as *Lactobacillus* and *Bifidobacterium*, may alleviate anxiety [61]. *Bifidobacterium (B.) longum*, *Bifidobacterium infantis*, *Lactobacillus (L.) helveticus*, and *L. rhamnosus* all had beneficial impacts on behavioral traits in anxiety animal models. Reversal of immunological pro-inflammatory factors or overexpression of GABA receptors were the mechanisms by which these effects were seen. In rats, long-term treatment of *Lactobacillus rhamnosus* reduced corticosterone levels and alleviated symptoms while also reversing alterations in GABA receptor levels in some brain areas. Mice with viral colitis and anxiety showed an improvement in anxiety-like behavior and a restoration of BDNF levels in their brains when supplemented with *B. longum* [68]. Strains of *Lactobacillus farciminis* reduced stress-induced leaky gut syndrome and hyperactive HPA axis activity in rats [69]. A combination of *Lactobacillus helveticus* (strain R0052) and *B. longum* (strain R0175) prevented stress-induced hypogenesis in the hippocampus regions of mice [70].

In addition, researchers found that injecting some strains of *Bifidobacterium* reduced stress- or depression-like behaviors. Probiotic mixtures of *Lactobacillus helveticus* and *B. longum* alleviated psychological distress in healthy human volunteers and showed anxiolytic effects in rats subjected to physiological stress conditions. The beneficial effects of *Lactobacillus* and *Bifidobacterium* strains in relieving gastrointestinal symptoms in children with autism spectrum disorder have been previously noted, and a new study builds on this. The research showed that the percentage of *Lactobacillus* bacteria was enhanced by a probiotic combination of *L. reuteri* and *B. longum*. The behavioral and gastrointestinal symptoms of people with autism spectrum disorder were significantly alleviated by a mixture of *Bifidobacterium*, *Streptococci*, and *Lactobacilli* [71,72]. Probiotic supplementation with *Lactobacillus rhamnosus* GG beginning in infancy reduced ADHD risk factors. According to research, adults whose early lives were marked by chronic stress may benefit from consuming Bifidobacterium, especially B. pseudocatenulatum CECT 7765 [73].

Prebiotics

Dietary components known as prebiotics promote the growth of beneficial bacteria that benefit the host's health. Prebiotics have beneficial effects on those with cognitive impairments, anxiety, and depression, according to research [74]. Patients with dementia, irritable bowel syndrome, and autism spectrum disorder have shown improved cognitive function, symptom relief, and overall health after taking prebiotics. An increase in beneficial species survival and a reduction in stress-induced HPA axis activation were seen in young, healthy adults who consumed dietary prebiotics such as Bimuno® galactooligosaccharides (BGOS) and non-digestible fructooligosaccharides. This was seen in a research that included 14,190 individuals. In rats that were exposed to extended mental stress, a prebiotic mixture consisting of fructooligosaccharide and galactooligosaccharide improved mood and alleviated anxiety and depression symptoms [75]. Injecting BGOS into rats reduced LPS-induced anxiety and depressive-like behavior. Decreased plasma tryptophan and corticosterone levels and increased 5-HT in the cecum are the mechanisms by which prebiotics ameliorate or prevent anxiety and depression [76,77]. Patients with irritable bowel syndrome who took BGOS for three months reported less anxiety and an overall better quality of life [78]. It also improved the rats' cognitive flexibility. Patients with irritable bowel syndrome who took short-chain fructooligosaccharides reported less anxiety and a higher concentration of bifidobacteria in their stool [79].

Synbiotics

Combining prebiotics with probiotics produces synbiotics, which benefit the host by increasing the number of beneficial bacteria in the digestive tract. Synbiotic pills, which include FOS and probiotics, were shown to enhance cognitive function and cause a small reduction in depressive symptoms in older research that lasted 24 weeks [80]. Subjects with severe depression showed a decrease in negative symptoms after taking a synbiotic mixture of Lactobacillus acidophilus T16, Bifidobacterium bifidum BIA-6, Bifidobacterium lactis BIA-7, and Bifidobacterium longum BIA-8, according to a randomized, double-blind clinical trial. In addition, BDNF serum levels were greater in the synbiotic combination group compared to the control group [81]. Autistic children showed enhanced resilience to gastrointestinal issues when given a mix of GOS, Limosilactobacillus (L.) reuteri, and Bifidobacterium (B.) longum. As demonstrated by reduced ammonium levels and increased short-chain fatty acid levels, this combination significantly improved the metabolism and activity of the gut microbiota. When people under stress took synbiotics, a mixture of *Lactobacillus paracasei* HII01, *Bifidobacterium animalis* subsp. *lactis*, galacto-oligosaccharides, and oligofructose, their negative feelings subsided. Modulating HPA axis activation and IL-10, IgA, and LPS production were the primary means of doing this. Administering symbiotic supplementation for 4 to 9 weeks led to a modest reduction in depressive symptoms, according to an extensive evaluation of scientific literature, which included clinical trials and observational research focused on patients with Major Depressive Disorder [82].

Dietary Modifications

The principal element impacting the variety and composition of gut microbiota is food, which includes eating habits, components, and composition. In contrast to control mice given standard chow, those given beef showed less anxiety, better memory, and a wider range of intestinal microbes. According to population surveys, psychological distress is less common among those who follow "traditional dietary practices" [83]. A similar study in India indicated that the percentage of Firmicutes was 34% greater than Bacteroidetes in healthy vegetarians [84,85]. Bacteroidetes account for 84% and Firmicutes for 4% in healthy persons who eat meat and other non-vegetarian foods, while the ratio is flipped in vegetarians. Alistipes, Bilophila, and Bacteroides are bile-tolerant microbes that are more prevalent on a non-vegetarian diet. Concurrently, it reduced the abundance of polysaccharide-degrading bacteria, such as *Roseburia*, *Firmicutes*, *Eubacterium rectale*, and *Ruminococcus bromii*.

Conversely, a healthy gut microbial composition has been associated with high-fiber diets (made up of plant-based foods, including fruits, nuts, and vegetables). One possible method for alleviating symptoms of autism spectrum disorder is to make dietary changes. As an example, research has demonstrated that the ketogenic diet characterized by a high-fat, moderate-protein, and extremely low-carbohydrate intake can ameliorate tantrums, eating disorders, and behavioral issues in children with autism spectrum disorder by influencing the expression of genes related to mitochondria [85]. Scientific studies have shown that following a ketogenic diet may significantly reduce seizure frequency while simultaneously improving behavioral symptoms, including improved cognitive abilities and social skills. Micronutrient supplementation was associated with a reduction in ADHD scores, according to a 10-week randomized controlled trial. An increase in the presence of Bifidobacterium was linked to this decline [86]. A gluten-free diet was shown to enhance behavior and elevate blood L-tryptophan levels, whereas a ketogenic diet was found to relieve the clinical symptoms of SZ [87,88].

A gluten- and casein-free diet, in conjunction with antibiotics and probiotics, offered modest therapeutic advantages in certain persons with schizophrenia, according to Latalova et al. [89]. According to studies, a high-fiber diet may lower colonic acidity and prevent the overgrowth of bad bacteria [90]. To maintain the diversity and abundance of gut microbiota throughout developmental stages marked by stress, it was helpful to supplement the diet with polyunsaturated fatty acids omega-3 and omega-6. Keeping the metabolic activity of Bifidobacterium and Lactobacillus intact while increasing their numbers allowed this to happen [91,92].

Transplantation of Fecal Microbiota

For many NPDs, FMT is the method of choice. This method entails utilizing endoscopy, an enema, and the oral delivery of frozen fecal contents to transfer complete or select microbial components from a healthy donor to the recipient. "Eubiosis" [93,94] refers to the process of establishing a diversified and healthy microbial community. In a study conducted on mice, it was shown that FMT from healthy persons reduced anxiety and depression symptoms after exposure to stressful situations. In individuals with irritable bowel syndrome and significant depression, the transplantation of fecal matter from a healthy donor alleviated depressive symptoms and anxiety. Clostridium difficile infection was also less common in the elderly as a result [94]. Separately, researchers looked at the effects of FMT, which involves transferring bacteria from depressed individuals to animals that have been treated with antibiotics and do not have a healthy gut microbiome. The findings showed that anxiety-like behaviors started to appear and that people no longer enjoyed things that they normally enjoyed. As shown in rats given FMT from a healthy donor, these effects were mainly explained by alterations in tryptophan metabolism [95]. The most effective and cost-effective approach to managing neurological and psychiatric disorders is functional magnetic therapy, which has few side effects.

## Conclusions

The human microbiome plays a critical role in health, particularly through gut bacteria. Dysbiosis, an imbalance in the microbiome, is linked to neurological disorders such as AD, PD, MS, stroke, and mental illnesses due to harmful microbial metabolites affecting brain function. FMT shows promise in restoring microbial balance and alleviating neurological symptoms. Gut microbiota signaling molecules, including hormones and SCFAs, influence CNS functions. PD and MS are associated with gut dysbiosis, with gastrointestinal issues often preceding motor symptoms in PD. MS is also linked to changes in gut microbiota, which influence brain pathways and offer potential therapeutic interventions through dietary changes, probiotics, and FMT. Dysbiosis has also been linked to ischemic stroke risk and psychological disorders such as AD and bipolar disorder. Future research is needed to further assess the therapeutic potential of FMT in mental and neurological health.
